# The importance of including uric acid in the definition of metabolic syndrome when assessing the mortality risk

**DOI:** 10.1007/s00392-021-01815-0

**Published:** 2021-02-18

**Authors:** Nicola Riccardo Pugliese, Alessandro Mengozzi, Agostino Virdis, Edoardo Casiglia, Valerie Tikhonoff, Arrigo F. G. Cicero, Andrea Ungar, Giulia Rivasi, Massimo Salvetti, Carlo M. Barbagallo, Michele Bombelli, Raffaella Dell’Oro, Berardino Bruno, Luciano Lippa, Lanfranco D’Elia, Paolo Verdecchia, Francesca Mallamaci, Massimo Cirillo, Marcello Rattazzi, Pietro Cirillo, Loreto Gesualdo, Alberto Mazza, Cristina Giannattasio, Alessandro Maloberti, Massimo Volpe, Giuliano Tocci, Georgios Georgiopoulos, Guido Iaccarino, Pietro Nazzaro, Gianfranco Parati, Paolo Palatini, Ferruccio Galletti, Claudio Ferri, Giovambattista Desideri, Francesca Viazzi, Roberto Pontremoli, Maria Lorenza Muiesan, Guido Grassi, Stefano Masi, Claudio Borghi, The Working Group on Uric Acid and Cardiovascular Risk of the Italian Society of Hypertension

**Affiliations:** 1 Department of Clinical and Experimental Medicine, University of Pisa, Via Roma, 67, 56126 Pisa, Italy; 2 Department of Medical and Surgical Science, Hypertension and Cardiovascular Risk Factors Research Center, Alma Mater Studiorum University of Bologna, Bologna, Italy; 3 Department of Clinical and Experimental Sciences, University of Brescia, Brescia, Italy; 4 Department of Geriatric and Intensive Care Medicine, Careggi Hospital and University of Florence, Florence, Italy; 5 Department of Internal Medicine, University of Genoa and Policlinico San Martino, Genoa, Italy; 6 Department of Life, Health and Environmental Sciences, University of L’Aquila, L’Aquila, Italy; 7 Italian Society of General Medicine, Avezzano, L’Aquila Italy; 8 Department of Cardiovascular, Neural and Metabolic Sciences, Istituto Auxologico Italiano, IRCCS S. Luca Hospital, Lucca, Italy; 9 Department of Medicine and Surgery, University of Milan-Bicocca, Milan, Italy; 10 Clinica Medica, Department of Medicine and Surgery, University of Milano-Bicocca, Monza, Italy; 11 Department of Clinical Medicine and Surgery, University of Naples ‘Federico II’, Naples, Italy; 12 Department of Advanced Biomedical Sciences, University of Naples ‘Federico II’, Naples, Italy; 13 Department of Public Health, University of Naples ‘Federico II’, Naples, Italy; 14 Department of Medicine and Studium Patavinum, University of Padua, Padua, Italy; 15 Department of Medicine, University of Padua, Padua, Italy; 16 Biomedical Department of Internal Medicine and Specialistics, University of Palermo, Palermo, Italy; 17 Hospital S. Maria Della Misericordia, Perugia, Italy; 18 Clinical Epidemiology of Renal Diseases and Hypertension, Reggio Cal Unit, CNR-IFC, Reggio Calabria, Italy; 19 Department of Medicine, Medicina Interna 1°, Ca’ Foncello University Hospital, University of Padova, Treviso, Italy; 20 Department of Emergency and Organ Transplantation–Nephrology, Dialysis and Transplantation Unit, Aldo Moro University of Bari, Bari, Italy; 21 Department of Internal Medicine, Hypertension Unit, General Hospital, Rovigo, Italy; 22 Cardiology IV, A. De Gasperis Department, Health Science Department, Niguarda Ca’ Granda Hospital, Milano-Bicocca University, Milan, Italy; 23 Hypertension Unit, Division of Cardiology, Department of Clinical and Molecular Medicine, Faculty of Medicine and Psychology, Sant’Andrea Hospital, University of Rome Sapienza, Rome, Italy; 24 IRCCS Neuromed, Pozzilli, IS Italy; 25 First Department of Cardiology, Medical School, Hippokration Hospital, University of Athens, Athens, Greece; 26 Department of Medical Basic Sciences, Neurosciences and Sense Organs, University of Bari Medical School, Bari, Italy

**Keywords:** Serum uric acid, Metabolic syndrome, Cardiovascular mortality, Prognosis

## Abstract

**Introduction:**

Serum uric acid (SUA) has been depicted as a contributory causal factor in metabolic syndrome (MS), which in turn, portends unfavourable prognosis.

**Aim:**

We assessed the prognostic role of SUA in patients with and without MS.

**Methods:**

We used data from the multicentre Uric Acid Right for Heart Health study and considered cardiovascular mortality (CVM) as death due to fatal myocardial infarction, stroke, sudden cardiac death, or heart failure.

**Results:**

A total of 9589 subjects (median age 58.5 years, 45% males) were included in the analysis, and 5100 (53%) patients had a final diagnosis of MS. After a median follow-up of 142 months, we observed 558 events. Using a previously validated cardiovascular SUA cut-off to predict CVM (> 5.1 mg/dL in women and 5.6 mg/dL in men), elevated SUA levels were significantly associated to a worse outcome in patients with and without MS (all *p* < 0.0001) and provided a significant net reclassification improvement of 7.1% over the diagnosis of MS for CVM (*p* = 0.004). Cox regression analyses identified an independent association between SUA and CVM (Hazard Ratio: 1.79 [95% CI, 1.15–2.79]; *p* < 0.0001) after the adjustment for MS, its single components and renal function. Three specific combinations of the MS components were associated with higher CVM when increasing SUA levels were reported, and systemic hypertension was the only individual component ever-present (all *p* < 0.0001).

**Conclusion:**

Increasing SUA levels are associated with a higher CVM risk irrespective of the presence of MS: a cardiovascular SUA threshold may improve risk stratification.

**Graphic abstract:**

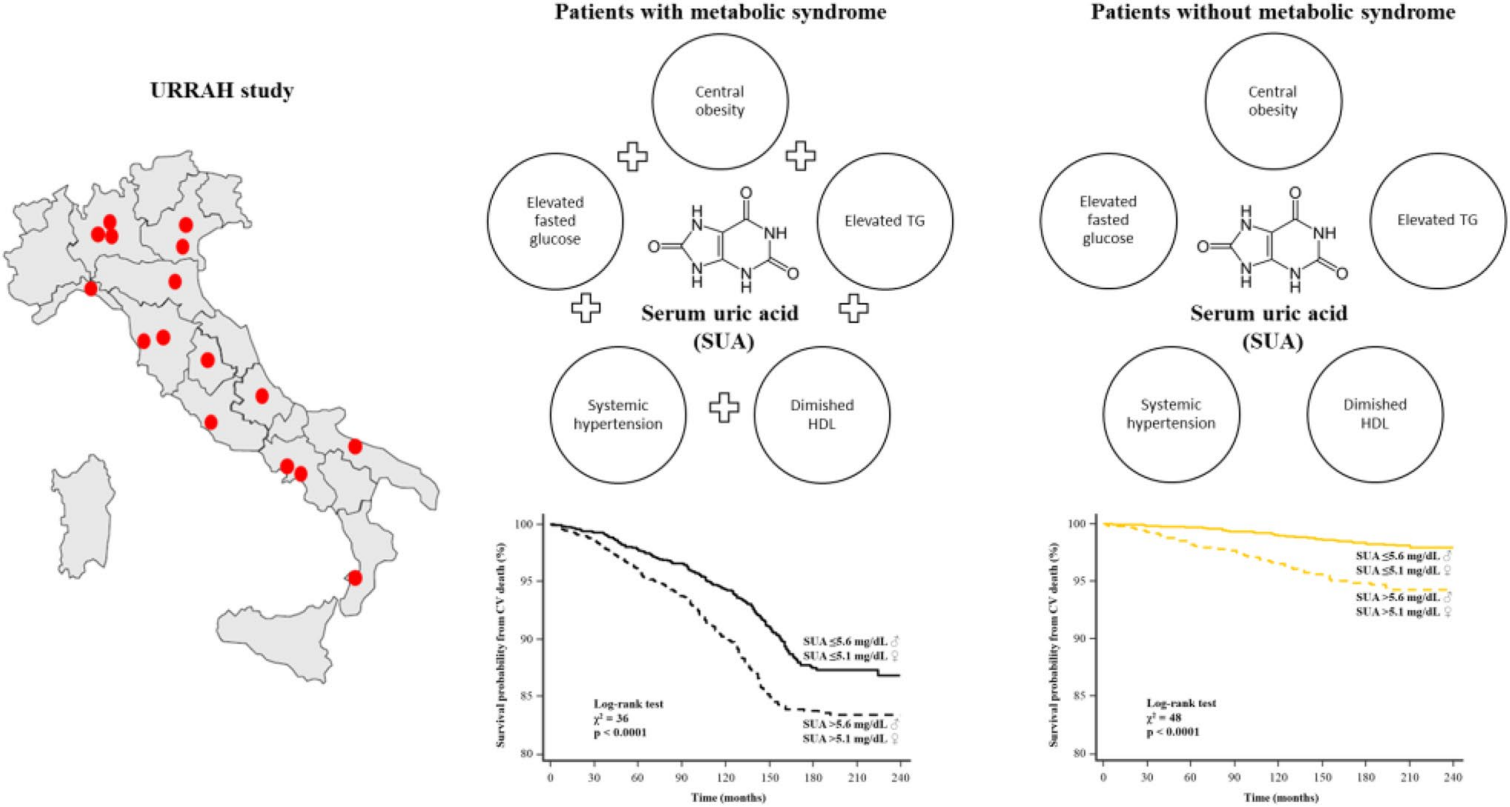

**Supplementary Information:**

The online version contains supplementary material available at 10.1007/s00392-021-01815-0.

## Introduction

Cardiovascular disease (CVD) remains the leading cause of morbidity and mortality worldwide, and risk prediction remains the cornerstone of preventive medicine [1–3]. The metabolic syndrome (MS) represents a constellation of risk factors for CVD [4], and it is associated with a twofold increase in the risk of CVD, cardiovascular mortality (CVM), myocardial infarction and stroke, and a 1.5-fold increase in the risk of all-cause mortality [5]. Increasing evidence suggests that uric acid may play a role in the development of MS, as hyperuricaemia belongs to a cluster of metabolic and haemodynamic abnormalities closely related to MS [4]. Several epidemiologic studies have reported a relation between serum uric acid (SUA) and CVD [6–9], as recognised by the latest European guidelines that recommend SUA evaluation in the stratification of the future cardiovascular risk of patients with arterial hypertension [3]. At the same time, little is known about the prognostic role of SUA in MS. The Working Group on SUA and cardiovascular risk of the Italian Society of Hypertension has specifically designed the URRAH project (Uric Acid Right for Heart Health) to study the relationship between SUA and CVD [10]. Using this extensive, prognostic registry, we investigated the role of SUA levels in improving further risk stratification of patients with MS. Moreover, we assessed in which combination of MS parameters the addition of SUA provided the greatest improvement in the prediction of CVM risk. This is important as it has the potential to provide clear indications to the physicians on the type of metabolic patient in whom the assessment of SUA might lead to the greatest improvement in the stratification of the risk of CVM and should be, therefore, evaluated. Finally, we questioned if a cardiovascular SUA cut-off value lower than that currently used in clinical practice to define the risk of gout could be functional as a prognostic parameter in addition to MS diagnosis.

## Methods

The Working Group on SUA and cardiovascular risk of the Italian Society of Hypertension has designed the URRAH project (Uric Acid Right for Heart Health) as a multicentre retrospective, observational cohort study, which involves data from several cohorts recruited within the Italian centres of hypertension and distributed in almost all the Italian regions. Datasets analysed during the current study are not publicly available but are available from the corresponding author on reasonable request.

### Data collection

As per protocol, SUA levels were collected from all the patients attending hypertension clinics at the time of the enrolment [10], together with information on cardiovascular risk factors where available. Anamnestic information, anthropometric measures (including waist circumference) and fasting blood lipid and glycaemic profiles were collected. Systolic and diastolic blood pressure was measured twice, in a quiet room, after five minutes resting and with the participant in sitting position. The second measure was used for all analyses. Hypertension was defined by the presence of at least two blood pressure recordings > 140/90 mmHg or treatment with antihypertensive medications. Diabetes mellitus was defined by treatment with antidiabetic drugs, fasting plasma glucose ≥ 126 mg/dL, or haemoglobin A1c ≥ 48 mmol/mol. Renal function was evaluated through estimation of the glomerular filtration rate (eGFR), according to the Chronic Kidney Disease Epidemiology Collaboration equation [11]. We assessed MS according to the 2001 third report of the Adult Treatment Panel from the National Cholesterol Education Program [12]. A definite diagnosis MS was assigned in the presence of three or more of the following five cardiovascular risk factors: (1) central obesity (waist circumference: men > 102 cm; women > 88 cm); (2) elevated triglycerides (≥ 150 mg/dL); (3) diminished high-density lipoprotein (HDL) cholesterol (men < 40 mg/dL; women < 50 mg/dL); (4) systemic hypertension (≥ 130/ ≥ 85 mm Hg); and (5) elevated fasting glucose (≥ 110 mg/dL). From the overall population of the URRAH project (*n* = 22,714), we selected only the subjects in which all the MS criteria were available (*n* = 9589).

### Outcomes

We considered cardiovascular mortality (CVM) at the end of the follow-up based on the following events: fatal events due to acute myocardial infarction, heart failure (HF) or stroke and sudden cardiac death. The definition of sudden cardiac death required documentation of significant arrhythmias or cardiac arrest. In case of death out of the hospital for which no autopsy was performed, sudden unexpected death was attributed to a cardiac cause. Information about death was obtained from hospital records or death certificates.

### Statistical analyses

All tests were two-sided, with a *p* value of < 0.05 considered significant. Data were analysed with SPSS version 25.0 (IBM Corp., Armonk, NY) and R 3.6.2 (R Foundation for Statistical Computing, Vienna, Austria). Normally distributed continuous variables were presented as mean ± SD and variables not following normal distribution as median (interquartile range). Differences in baseline characteristics were evaluated by independent samples *t* test or Mann–Whitney *U* test for continuous variables and *χ*^2^ test for nominal variables.

### Survival analyses

Clinical follow-up data were censored at the time of the last visit or, for patients lost during follow-up, at the last date they were known to be alive. Kaplan–Meier survival curves for patients with and without MS were generated, and log-rank tests were used to assess differences between curves. Multivariable Cox proportional-hazards model was used to examine the association between MS and the outcome, including with stepwise forward selection SUA levels and all available clinical variables with biological plausibility selection (entry and removal value of *p* < 0.01 and *p* < 0.10, respectively). We tested interactions of SUA with age, gender (male), diabetes mellitus, eGFR and diuretics by incorporating corresponding interaction terms in the analysis. Associations are presented as hazard ratio (HR) with 95% *Cis* and unstandardised β-regression coefficients (HR for CVM is relative to a one-unit change in the continuous variables included in the model). Variance inflation factor > 5 was used to exclude multi-collinearity between selected variables. The association between different SUA levels and the outcome in patients with and without MS was analysed using restricted cubic splines with three knots and a reference SUA level of 4.1–5 mg/dL. Analyses were adjusted for the covariates that were significant at the previous Cox regression to produce a smooth curve vs HR for CVM in the *y*-axis. We assessed the prognostic accuracy using the receiver operating characteristic (ROC) to calculate the area under the curve (AUC) and the cut-off point with the highest Youden index. As MS is defined by the presence of at least three out of five cardiovascular risk factors, 16 combinations are possible using binomial coefficients:

$$\frac{5!}{{\left( {5 - 3} \right)! \cdot 3!}} + \frac{5!}{{\left( {5 - 4} \right)! \cdot 4!}} + \frac{5!}{{\left( {5 - 4} \right)! \cdot 5!}}$$

Therefore, we tested the interaction between each MS combination and SUA levels in predicting adverse events, to identify the MS combinations that are more associated with CVM when SUA is added to the model.

### Reclassification and discrimination analysis

For estimating measures of reclassification and discrimination, we estimated the added value of identifying elevated SUA levels to predict the CVM using a model based on the presence of MS. Reclassification was deemed appropriate for participants with adverse outcome during clinical follow-up moving up in risk category and for participants without event moving down in risk category. Reclassification was summarised using continuous net reclassification index (cNRI) and integrated discrimination improvement (IDI) [13].

## Results

The baseline characteristics of the studied population are reported in Table 1. A definite diagnosis of MS was observed in 5100/9589 (53%) patients, which on average were older and more overweight, with a significantly higher frequency of the main CV risk factors and co-morbidities. As expected, blood tests showed a worse glycaemic and lipid profile in the subjects with MS. The most commonly prescribed medications were angiotensin receptor blockers, angiotensin-converting enzyme inhibitors, and dihydropyridine calcium channel blockers. Overall, drug therapy was more intense in those with MS. Supplemental Table 1 [Pugliese, Nicola Riccardo (2021): Supplemental material_rebuttal. figshare. Media. https://doi.org/10.6084/m9.figshare.13635062.v1] presents the same clinical characteristics as the population stratified by the previously validated cut-off of SUA levels for predicting CVM (♂ > 5.6 mg/dL; ♀ > 5.1 mg/dL)[14].

**Table 1:** Population characteristics in patients with and without metabolic syndrome (MS)

Variable	MS (*n* = 5100)	Non-MS (*n* = 4489)	*p* value
Demographics			
Age, years	62 ± 13	52 ± 16	** < 0.0001**
Male	1936 (38)	2351 (52)	** < 0.0001**
BMI, kg/m^2^	29.1 ± 4.3	25.3 ± 4.1	** < 0.0001**
Waist circumference, cm	100 ± 11	86 ± 12	** < 0.0001**
Family history of arterial hypertension	2805 (55)	2245 (50)	** < 0.0001**
Family history of CVD	1938 (38)	2200 (49)	** < 0.0001**
Current smoker	1071 (21)	1077 (24)	** < 0.0001**
Clinical evaluation			
Heart rate, beats/min	72 ± 12	67 ± 11	0.2
Systolic blood pressure, mmHg	153 ± 21	131 ± 21	** < 0.0001**
Diastolic blood pressure, mmHg	88 ± 12	78 ± 11	** < 0.0001**
Arterial Hypertension	4405 (86)	1985 (44)	** < 0.0001**
Diabetes mellitus	1136 (22)	165 (4)	** < 0.0001**
CKD	945 (19)	323 (7)	** < 0.0001**
Gout	90 (2)	5 (0.1)	** < 0.0001**
Blood tests			
Haemoglobin, g/dL	14.6 ± 1.4	14.2 ± 1.5	** < 0.0001**
Haematocrit, %	43 ± 4	43 ± 4	0.5
Total cholesterol, mg/dL	217 ± 40	210 ± 40	** < 0.0001**
HDL, mg/dL	43 ± 12	57 ± 15	** < 0.0001**
Triglycerides, mg/dL	176 (138 – 227)	85 (63 – 115)	** < 0.0001**
Creatinine, mg/dL	0.98 ± 0.2	0.88 ± 0.2	** < 0.0001**
eGFR, mL/min/1.73 m^2^	69 ± 20	84 ± 21	** < 0.0001**
Fasting blood sugar, mg/dL	114 ± 35	88 ± 15	** < 0.0001**
Serum uric acid, mg/dL	5.7 ± 1.4	4.8 ± 1.2	** < 0.0001**
Elevated serum uric acid^a^	2599 (51)	820 (18)	** < 0.0001**
Azotemia, mg/dL	35 ± 11	27 ± 8	** < 0.0001**
Therapy			
ACE inhibitor	3315 (65)	1571 (35)	** < 0.0001**
Angiotensin receptor blocker	3519 (69)	1392 (31)	** < 0.0001**
DHP CCB	663 (13)	225 (5)	** < 0.0001**
Non-DHP CCB	51 (1)	45 (1)	0.8
Beta-Blocker	612 (12)	359 (8)	** < 0.0001**
Allopurinol	79 (1.5)	14 (0.3)	** < 0.0001**
Statins	314 (7)	412 (8)	0.05
Diuretics	605 (12)	285 (6)	** < 0.0001**
Hydrochlorothiazide	226 (12)	135 (5)	** < 0.0001**
Indapamide	37 (2)	33 (1)	**0.02**
Chlortalidone	36 (2)	27 (0.6)	** < 0.0001**
Loop diuretics	306 (6)	90 (2)	** < 0.0001**

Values are mean ± standard deviation, *n* (%), or median [25th quartile, 75th quartile]

*ACE* angiotensin-converting enzyme, *BMI* body mass index, *CKD* chronic kidney disease (eGFR < 60 mL/min/1.73 m^2^), *CVD* cardiovascular disease, *DHP CCB* dihydropyridine calcium channel blocker, *eGFR* estimated glomerular filtration rate, *HDL* high-density lipoprotein, *SUA* serum uric acid

^a^♂ > 5.6 mg/dL; **♀** > 5.1 mg/dL

*p* values in bold indicate numbers that are significant on the 95% confidence limit

**Fig. 1:**
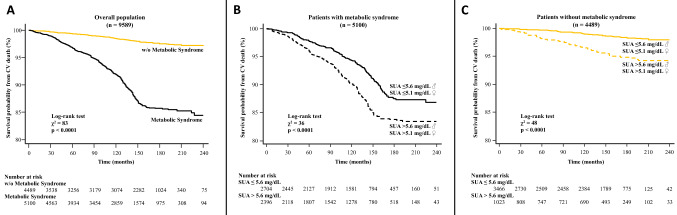
Kaplan–Meier survival curves for cardiovascular death after a median follow-up of 18.5 months in the overall population (**a**), and patients with and without metabolic syndrome (**b**, **c**, respectively). The patients are stratified according to serum uric acid into low (♂ ≤ 5.6 mg/dL; ♀ ≤ 5.1 mg/dL) and elevated levels (♂ > 5.6 mg/dL; ♀ > 5.1 mg/dL)

**Fig. 2:**
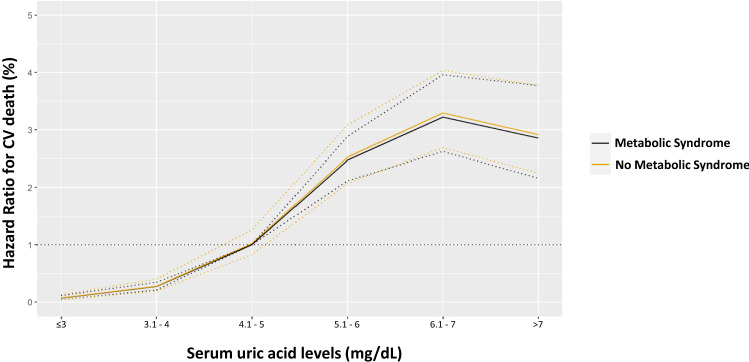
Hazard ratio for cardiovascular (CV) death across the range of the serum uric acid levels (reference value 4.1–5 mg/dL) in patients with and without metabolic syndrome. Estimates are from proportional hazard modelling as a restricted cubic spline function of SUA levels and dotted lines show 95% confidence intervals. Analyses were adjusted for age, gender, arterial hypertension, diabetes mellitus and statin intake

During a median follow-up time of 142 months (interquartile range 60–163), a total of 558 CV deaths were recorded (6%), of which 175 were due to fatal myocardial infarction. Any adverse event was observed more frequently in patients with MS (Table 2), as confirmed by Kaplan–Meier analysis (Fig. 1a). The survival probability free from CVM for elevated and low SUA in the overall population was 87% and 94%, respectively. The same analysis was conducted in patients with and without MS, showing high levels of SUA resulted in a worse outcome in both groups (log-rank test: all *p* < 0.0001; Fig. 1b, c). At the univariable Cox model, MS was associated with an increased risk of CVM (HR 5.21, 95% CI 4.14–6.54; *p* < 0.0001). This association remained highly significant even in a multivariable model adjusted for MS individual components, SUA levels, multiple CV risk factors and therapy: HR 2.25, 95% CI 1.69–2.99); *p* < 0.001 (Table 3). Five interaction terms were tested [SUA * age, SUA * gender (male), SUA * diabetes mellitus, SUA * eGFR and SUA * diuretics], and only SUA * age was significant when included in the model (HR 0.98, 95% CI 0.97–0.99; *p* = 0.03). Then, we confirmed the relation between MS, SUA and outcome after subdividing the population according to age [≥ and < 65 years; Supplemental Tables 2–3, Pugliese, Nicola Riccardo (2021): Supplemental material_rebuttal. figshare. Media. https://doi.org/10.6084/m9.figshare.13635062.v1]. Likewise, when excluding 191 participants taking allopurinol, the results were not substantively different from the overall analysis [Supplemental Table 4, Pugliese, Nicola Riccardo (2021): Supplemental material_rebuttal. figshare. Media. https://doi.org/10.6084/m9.figshare.13635062.v1]. Plotting the estimates from proportional-hazard modelling against SUA levels (reference level 4.1–5 mg/dL) in patients with and without MS shows overlapping relative risks that increase for SUA > 5 mg/dL and reach a plateau for values > 7 (Fig. 2). Also, ROC analysis showed the prognostic accuracy of SUA was similar in patients with and without MS (AUC: 0.669 vs 0.694; *p* = 0.1) but requiring different cut-offs (6.3 vs 5.6 mg/dL).

**Table 2:** Clinical follow-up: cumulative event rates and Hazard Ratio for cardiovascular mortality in patients with and without metabolic syndrome

Event	MS (*n* = 5100)	Non-MS (*n* = 4489)	Hazard ratio (95% CI)*	*p* value*
Cardiovascular death	430 (8.4)	128 (2.9)	2.96 (2.56–3.33)	** < 0.0001**
Fatal myocardial infarction	140 (2.7)	35 (0.8)	2.87 (1.75–5.59)	** < 0.0001**
Fatal stroke	105 (2.1)	38 (0.8)	2.15 (1.49–4.82)	** < 0.0001**
Sudden cardiac death	58 (1.1)	25 (0.6)	1.57 (1.19–3.76)	**0.01**
End-stage heart failure	127 (2.5)	30 (0.7)	3.49 (2.67–5.77)	** < 0.0001**

Legend as in the previous tables

*The hazard ratio is for the MS group as compared with the Non-MS group, and *p* values were calculated by the log-rank test and are unadjusted for multiple variables

*p* values in bold indicate numbers that are significant on the 95% confidence limit

**Table 3:** Stepwise Cox proportional-hazards analysis for cardiovascular death

Variable	Hazard ratio (95% CI)	*p* value	*β* regression coefficient
Age, years	1.14 (1.09–1.18)	** < 0.0001**	0.1
Male	1.44 (1.19–1.73)	**0.01**	0.4
Arterial Hypertension	1.59 (1.17–2.15)	**0.003**	0.5
Diabetes mellitus	2.74 (1.33–5.65)	**0.001**	1.1
Serum uric acid, mg/dL	1.79 (1.15–2.79)	** < 0.0001**	0.6
Metabolic syndrome	2.25 (1.69–2.99)	** < 0.0001**	0.8
Statins	0.33 (0.21–0.52)	**0.001**	− 1.1
SUA * age	0.98 (0.97–0.99)	**0.03**	0.01
BMI, kg/m^2^	0.97 (0.95–1.02)	0.1	–
Current smoker	1.18 (0.89–1.23)	0.2	–
eGFR, mL/min/1.73 m^2^	1.17 (0.89–1.29)	0.2	–
Gout	1.34 (0.48–2.97)	0.5	–
Haemoglobin, g/dL	1.19 (0.88–1.26)	0.2	–
Haematocrit, %	1.08 (0.75–2.84)	0.3	–
Total cholesterol, mg/dL	1.01 (0.99–1.02)	0.3	–
HDL, mg/dL	0.99 (0.98–1.01)	0.1	–
Triglycerides, mg/dL	1.01 (0.99–1.01)	0.5	–
Diuretics	1.43 (0.82–3.63)	0.1	–
SUA * gender (male)	1.04 (0.93–1.17)	0.1	–
SUA * diabetes mellitus	0.95 (0.85–1.08)	0.4	–
SUA * eGFR	0.87 (0.79–1.14)	0.5	–
SUA * diuretics	1.05 (0.91–1.22)	0.5	–

Legend as in the previous tables

*p* values in bold indicate numbers that are significant on the 95% confidence limit

Eight of the 16 combinations were representative of most of the cases of MS (4272/5100, 84%) and the most frequents were systemic hypertension + elevated triglycerides + diminished HDL (885/5100, 17%) and systemic hypertension + elevated triglycerides + elevated fasting glucose (843/5100, 16%). At Cox regression analysis for predicting CVM, the previous two combinations along with systemic hypertension + diminished HDL + elevated fasting glucose (565/5100, 11%) showed the highest β regression coefficients after the interaction with SUA levels: 0.08 ± 0.03, 0.16 ± 0.03 and 0.18 ± 0.03 (all *p* < 0.0001).

**Fig. 3:**
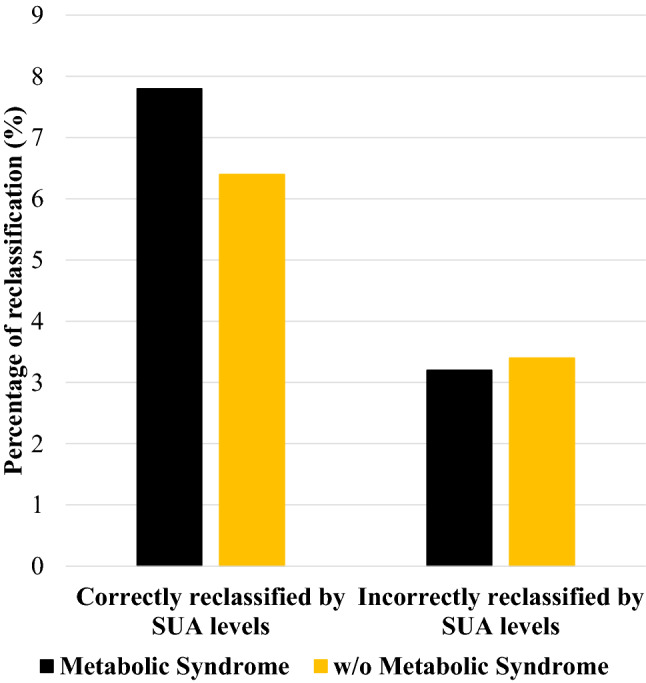
Reclassification analysis. Percentage of patients with and without metabolic syndrome correctly and incorrectly reclassified for event risk prediction when elevated serum uric acid (SUA) levels (♂ > 5.6 mg/dL; ♀ > 5.1 mg/dL) were added to the model

The addition of sex-specific elevated SUA levels (♂ > 5.6 mg/dL; ♀ > 5.1 mg/dL) to a model based on the presence of MS improved CVM classification: 47/588 patients with events (8%) were reclassified correctly, while 11 (2%) were reclassified incorrectly. At the same time, 632/9031 (7%) patients without adverse outcome (i.e., CVM) underwent appropriate reclassification, while 305 (3%) were reclassified inappropriately. In particular, reclassification analysis correctly reclassified 7.8% and 6.4% of subjects without and with MS, yielding a cNRI of 7.1%, *p* = 0.004 (Fig. 3). Discrimination was also improved, using the same cut-points, as indicated by IDI: (4.6%, *p* = 0.001). Reclassification analysis was further tested in different models, each based on the presence of the three MS combinations showing the most significant association with CVM after the interaction with SUA levels. Elevated SUA levels (♂ > 5.6 mg/dL; ♀ > 5.1 mg/dL) improved CVM reclassification (i.e., NRI) and discrimination (i.e., IDI) in all the models [Supplemental Table 5, Pugliese, Nicola Riccardo (2021): Supplemental material_rebuttal. figshare. Media. https://doi.org/10.6084/m9.figshare.13635062.v1]. The most significant improvement in predicting CVM was noted in MS combination including systemic hypertension + elevated triglycerides + diminished HDL (cNRI = 8.3%, *p* = 0.003; IDI = 5.1%; *p* = 0.001). We tested the correlation analyses between SUA levels and the individual components of MS [Supplemental Table 6, Pugliese, Nicola Riccardo (2021): Supplemental material_rebuttal. figshare. Media. https://doi.org/10.6084/m9.figshare.13635062.v1] and observed a significant correlation with all the variables (all *p* < 0.0001): the highest correlation coefficient was observed with fasting glucose (*r* = 0.41) and triglycerides (*r* = 0.31).

## Discussion

The present study suggests that the information on SUA levels refines the prediction of CVM obtained with MS and might help discrimination and reclassification of subjects at higher and lower CV risk [5].

The pathophysiological mechanism by which MS increases cardiovascular risk remains under debate [15]. Insulin resistance and central obesity are postulated to be the critical components of the metabolic syndrome, and both lead to glucose intolerance and dysglycemia [5]. Historically, elevated SUA levels in MS has been attributed to hyperinsulinemia, since insulin reduces renal excretion of uric acid [16]. Hyperuricemia, however, often precedes the development of hyperinsulinemia [17], obesity [18], and diabetes [19]. Independently on the cause-consequence relationship between SUA and MS, the presence of SUA in patients with MS seems to mark a greater risk of CVM. Indeed, animal models have shown that decreasing uric acid levels can prevent or reverse MS features [20], probably because hyperuricemia can induce endothelial dysfunction [21] and/or oxidative changes in adipocytes [22], which are typical stigmata of MS. Interestingly, all these features are increasingly described in HF with preserved ejection fraction (HFpEF), pointing to the existence of an inflammatory-metabolic phenotype [23]. A recent subanalysis from the PARAGON-HF trial demonstrated that hyperuricaemia was associated with an increased risk of adverse cardiovascular outcomes (CVM and HF hospitalisation) [24], suggesting that SUA may be a relevant therapeutic target also in HFpEF.

Our previous analysis documented that SUA levels are associated with all-cause mortality and CVM in hypertensive patients, independently of other CVD risk factors and the validated Heart Score risk algorithm. Also, the association of SUA with all-cause mortality and CVM was continuous [14]. The present analysis focuses on the role of uric acid in patients with MS, which represent a population at high risk of adverse CV events [5]. Krishnan et al. have analysed the incidence of myocardial infarction in 12,866 men at high risk of adverse coronary events and found a significant risk relationship with hyperuricemia (≥ 7 mg/dL) that was independent of renal function, diuretic use and MS [25]. The present study provides further insights on the topic, because we proved that higher SUA level portends a worse outcome regardless of the presence of MS or other common CV factors, including interaction with diuretic intake. Noteworthy, we enrolled a population with an intermediate risk of adverse CV events according to Heart Score risk charts. Nevertheless, when we analysed SUA levels as a continuous variable in MS and non-MS, we observed overlapping risk curves that raise when SUA > 5 mg/dL and reach a plateau for values > 7 mg/dl. Indeed, the prognostic accuracy of SUA levels was similar in the two groups, supporting the use of a cardiovascular threshold of SUA [14] to improve discrimination and reclassification of CVM in patients with and without MS.

### Clinical perspectives

The present study confirms the importance of implementing SUA dosage in clinical practice for a more precise CV risk stratification also in patients with MS, of which hyperuricemia was formerly a part [26]. In particular, we identified some specific combinations of the MS individual components associated with significantly higher CVM when increasing SUA levels are reported, i.e., systemic hypertension with two between elevated triglycerides, diminished HDL and elevated fasting glucose. Interestingly, the most significant improvement in predicting CVM was noted in the most common MS combination (i.e., systemic hypertension + elevated triglycerides + diminished HDL), encouraging the adoption of a cardiovascular threshold of SUA in clinical practice. As several Mendelian randomisation studies have suggested that the association between SUA and CVM is likely to be influenced by pleiotrophy [27–29], understanding the complex relationship between SUA and other CVD risk factor is critical. Moreover, it might help the identification of patients who could experience the most significant benefits from urate-lowering treatments and, as such, refine the inclusion and exclusion criteria in randomised clinical trials [26]. Indeed, a careful assessment of the clinical characteristics of the patients included in previous trials reveals a very heterogeneous population. As an example, most of the patients included in the CARES trial were obese [30], while the FREED trial enrolled mainly normal-weight subjects with a significantly lower proportion of hyperlipemia [31, 32]. Clearer identification of the patient phenotype in which SUA might have a greater impact on mortality could avoid further confusion in the future. Noteworthy, the risk of CVM may increase with SUA levels lower than those currently used in clinical practice to define the risk of gout [24]. This is of utmost importance, since there is an urgent need to develop and implement prevention and treatment strategies (e.g., lifestyle programs, diets, and pharmacotherapies) to reduce the CV burden. Prospective clinical trials are advisable to investigate whether the benefit of lowering SUA based on a cardiovascular threshold portends any prognostic advantage.

### Limitations

The present findings relied on a large sample size and extended follow-up, which enabled the accumulation of sufficient events for robust and reliable analysis, including hard endpoints. However, the study design is retrospective. As such, the results should be cautiously interpreted, because the risk of selection bias is high, and there might be unmeasured variables that could potentially influence the relationship between SUA levels and outcomes. As this limitation is common to many other reports analysing the relationship between SUA and CVM, prospective studies are much needed, including randomised trials with urate-lowering drugs. Dietary intake assessment was not implemented in our study, and thus, we had no information about the type of food consumed that may affect SUA levels. The patients enrolled had relatively low levels of SUA (only 1157/9589 [12%] had SUA > 7 mg/dL); therefore, our findings might underestimate the mortality risk in populations with higher SUA levels. We did not collect data about non-fatal events during the clinical follow-up. The URRAH study was composed of subjects of white ethnicity, which included a percentage of patients selected from Hypertension clinics, thus resulting in a heterogeneous population. Consequently, further studies are needed to confirm that the thresholds of SUA emerging from our analyses are valid also in other populations.

## Conclusions

Results from the present study confirm that sex-specific SUA cut-offs lower than those commonly considered to be at risk of gout are significantly associated with an increased risk of CVM in patients with MS, and independently from other conventional cardiovascular risk factors. If confirmed by future prospective studies, our data might contribute to improving further risk discrimination and reclassification of subjects with MS, which are already at high risk of CVM.

## Supplementary Information

Below is the link to the electronic supplementary material.

Supplementary file1 (DOCX 34 KB)

## Data Availability

Datasets generated during and/or analysed during the current study are not publicly available but are available from the corresponding author on reasonable request.
