# Supplementary material for: The importance of including uric acid in the definition of metabolic syndrome when assessing the mortality risk
Source: Clin Res Cardiol. 2021 Feb 18;110(7):1073–82. doi: 10.1007/s00392-021-01815-0 (PMC8238697; doi:10.1007/s00392-021-01815-0)
Supplement: Supplementary file 1 — Supplementary file1 (DOCX 34 KB) [file 392_2021_1815_MOESM1_ESM.docx]

**Supplemental Table 1.** Population characteristics in patients with low SUA vs elevated SUA (♂ > 5.6 mg/dL; **♀** > 5.1 mg/dL).

| Variable | Low SUA  (n=6170) | Elevated SUA  (n=3419) | p-value |
| --- | --- | --- | --- |
| Demographics |  |  |  |
| Age, years | 56±16 | 60±15 | **<0.0001** |
| Male | 2530 (41) | 1757 (49) | **<0.0001** |
| BMI, Kg/m^2^ | 26.5±4.6 | 28.6±4.3 | **<0.0001** |
| Waist circumference, cm | 88.4±13.3 | 97.9±11.6 | **<0.0001** |
| Family history of arterial hypertension | 3208 (52) | 1812 (53) | 0.3 |
| Family history of CVD | 2776 (45) | 1770 (43) | 0.1 |
| Current smoker | 1357 (22) | 821 (24) | **0.04** |
| Clinical evaluation |  |  |  |
| Heart rate, beats/min | 70±12 | 70±13 | 0.2 |
| Systolic blood pressure, mmHg | 140±24 | 148±23 | **<0.0001** |
| Diastolic blood pressure, mmHg | 82±12 | 86±13 | **<0.0001** |
| Arterial Hypertension | 3713 (60) | 2677 (78) | **<0.0001** |
| Diabetes mellitus | 740 (12) | 581 (17) | **<0.0001** |
| CKD | 617 (10) | 650 (19) | **<0.0001** |
| Gout | 37 (0.6) | 68 (2) | **<0.0001** |
| Blood tests |  |  |  |
| Haemoglobin, g/dL | 14±1 | 15±1 | **<0.0001** |
| Haematocrit, % | 43±4 | 44±4 | **<0.0001** |
| Total cholesterol, mg/dL | 212±40 | 217±40 | **<0.0001** |
| HDL, mg/dL | 52±15 | 46±13 | **<0.0001** |
| Triglycerides, mg/dL | 108 (73 – 167) | 159 (108 – 216) | **<0.0001** |
| Creatinine, mg/dL | 0.88±0.2 | 1.04±0.3 | **<0.0001** |
| eGFR, mL/min/1.73 m^2^ | 81±21 | 68±20 | **<0.0001** |
| Fasting blood sugar, mg/dL | 98±31 | 105±27 | **<0.0001** |
| Serum uric acid, mg/dL | 4.4±0.8 | 6.8±0.9 | **<0.0001** |
| Azotemia, mg/dL | 30±10 | 34±12 | **<0.0001** |
| Metabolic syndrome | 2501 (41) | 2599 (76) | **<0.0001** |
| Waist circumference (♂ > 101.6 cm; ♀ > 88.9 cm) | 2159 (35) | 1949 (57) | **<0.0001** |
| Blood pressure >130/85 mmHg | 4072 (66) | 2804 (82) | **<0.0001** |
| Fasting blood sugar >100 mg/dL | 1844 (30) | 1813 (53) | **<0.0001** |
| HDL (♂ < 40 mg/dL; ♀ < 50 mg/dL) | 2523 (41) | 1982 (58) | **<0.0001** |
| Triglycerides > 150 mg/dL | 1967 (32) | 1984 (58) | **<0.0001** |
| Therapy |  |  |  |
| ACE inhibitor | 864 (14) | 650 (19) | **<0.0001** |
| Angiotensin receptor blocker | 555 (9) | 513 (15) | **<0.0001** |
| DHP CCB | 432 (7) | 410 (12) | **<0.0001** |
| Non-DHP CCB | 66 (1) | 34 (1) | 0.5 |
| Beta-Blocker | 555 (9) | 446 (13) | **<0.0001** |
| Allopurinol | 123 (2) | 68 (2) | 0.4 |
| Statins | 432 (7) | 307 (9) | **<0.0001** |
| Diuretics | 625 (10) | 818 (24) | **<0.0001** |
| Hydrochlorothiazide | 370 (6) | 410 (12) | **<0.0001** |
| Indapamide | 67 (1) | 67 (2) | **0.005** |
| Chlortalidone | 66 (1) | 102 (3) | **<0.0001** |
| Loop diuretics | 122 (2) | 239 (7) | **<0.0001** |

Values are mean ± standard deviation, n (%), or median [25th quartile, 75th quartile].

ACE: angiotensin-converting enzyme; BMI: body mass index; CKD: chronic kidney disease (eGFR< 60 mL/min/1.73 m^2^); CVD: cardiovascular disease; DHP CCB: dihydropyridine calcium channel blocker; eGFR: estimated glomerular filtration rate; HDL: high-density lipoprotein; SUA: serum uric acid.**Supplemental Table 2.** Stepwise Cox proportional-hazards analysis for cardiovascular death in patient with <65 years old (n=5985).

| Variable | Hazard ratio  (95% CI) | p-value | β Regression coefficient |
| --- | --- | --- | --- |
| Male | 1.86 (1.23 – 2.81) | **0.003** | 0.6 |
| Arterial Hypertension | 4.07 (2.11 – 7.83) | **<0.0001** | 1.4 |
| Diabetes mellitus | 4.11 (2.61 – 6.49) | **<0.0001** | 1.4 |
| Serum uric acid, mg/dL | 1.19 (1.04 – 1.36) | **0.01** | 0.2 |
| Metabolic syndrome | 2.08 (1.19 – 3.63) | **0.01** | 0.7 |
| Statins | 0.77 (0.32 – 1.19) | 0.1 | - |
| BMI, Kg/m^2^ | 0.94 (0.95 – 1.03) | 0.1 | - |
| Current smoker | 1.17 (0.88 – 1.25) | 0.2 | - |
| eGFR, mL/min/1.73 m^2^ | 1.38 (0.79 – 2.16) | 0.3 | - |
| Gout | 1.32 (0.44 – 3.05) | 0.5 | - |
| Haemoglobin, g/dL | 1.10 (0.83 – 1.22) | 0.3 | - |
| Haematocrit, % | 1.16 (0.77 – 2.83) | 0.3 | - |
| Total cholesterol, mg/dL | 1.01 (0.99 – 1.01) | 0.2 | - |
| HDL, mg/dL | 0.99 (0.98 – 1.01) | 0.1 | - |
| Triglycerides, mg/dL | 1.01 (0.99 – 1.01) | 0.4 | - |
| Diuretics | 1.75 (0.75 – 4.08) | 0.1 | - |
| SUA*gender (male) | 1.06 (0.92 – 1.24) | 0.2 |  |
| SUA*diabetes mellitus | 0.97 (0.82 – 1.14) | 0.4 | - |
| SUA*eGFR | 0.87 (0.75 – 1.13) | 0.4 | - |
| SUA*diuretics | 1.01 (0.89 – 1.21) | 0.5 | - |

Legend as in the previous tables.

**Supplemental Table 3.** Stepwise Cox proportional-hazards analysis for cardiovascular death in patients with ≥65 years old (n=3604).

| Variable | Hazard ratio  (95% CI) | p-value | β Regression coefficient |
| --- | --- | --- | --- |
| Diabetes mellitus | 2.11 (1.74 – 2.55) | **<0.0001** | 0.7 |
| Serum uric acid, mg/dL | 1.12 (1.06 – 1.19) | **<0.0001** | 0.1 |
| Metabolic syndrome | 2.11 (1.62 – 2.76) | **<0.0001** | 0.7 |
| Statins | 0.34 (0.21 – 0.54) | **<0.0001** | -1.1 |
| Male | 1.14 (0.81 – 1.36) | 0.1 | - |
| Arterial Hypertension | 1.39 (0.93 – 1.74) | 0.1 | - |
| BMI, Kg/m^2^ | 0.94 (0.95 – 1.03) | 0.1 | - |
| Current smoker | 1.13 (0.88 – 1.22) | 0.2 | - |
| eGFR, mL/min/1.73 m^2^ | 1.23 (0.84 – 2.09) | 0.3 | - |
| Gout | 1.32 (0.44 – 3.05) | 0.5 | - |
| Haemoglobin, g/dL | 1.10 (0.83 – 1.21) | 0.3 | - |
| Haematocrit, % | 1.15 (0.77 – 2.83) | 0.3 | - |
| Total cholesterol, mg/dL | 1.06 (0.99 – 1.01) | 0.2 | - |
| HDL, mg/dL | 0.95 (0.98 – 1.01) | 0.1 | - |
| Triglycerides, mg/dL | 1.01 (0.99 – 1.01) | 0.4 | - |
| Diuretics | 1.75 (0.75 – 4.07) | 0.1 | - |
| SUA*gender (male) | 1.05 (0.91 – 1.19) | 0.2 |  |
| SUA*diabetes mellitus | 0.96 (0.82 – 1.14) | 0.4 | - |
| SUA*eGFR | 0.77 (0.63 – 1.35) | 0.6 | - |
| SUA*diuretics | 1.09 (0.89 – 1.22) | 0.5 | - |

Legend as in the previous tables.

**Supplemental Table 4.** Stepwise Cox proportional-hazards analysis for cardiovascular death after excluding patients taking allopurinol.

| Variable | Hazard ratio  (95% CI) | p-value | β Regression coefficient |
| --- | --- | --- | --- |
| Age, years | 1.09 (1.08 – 1.10) | **<0.0001** | 0.2 |
| Male | 1.33 (1.11 – 1.58) | **0.001** | 0.3 |
| Arterial Hypertension | 1.52 (1.13 – 2.04) | **0.005** | 0.5 |
| Diabetes mellitus | 3.04 (1.56 – 5.93) | **0.001** | 1.1 |
| Serum uric acid, mg/dL | 1.19 (1.05 – 1.35) | **0.007** | 0.2 |
| Metabolic syndrome | 2.41 (1.88 – 3.09) | **<0.0001** | 0.9 |
| Statins | 0.32 (0.21 – 0.49) | **0.001** | -1.1 |
| BMI, Kg/m^2^ | 0.96 (0.95 – 1.03) | 0.1 | - |
| Current smoker | 1.10 (0.88 – 1.25) | 0.2 | - |
| eGFR, mL/min/1.73 m^2^ | 1.19 (0.91 – 1.22) | 0.3 | - |
| Gout | 1.37 (0.44 – 3.05) | 0.5 | - |
| Haemoglobin, g/dL | 1.11 (0.83 – 1.21) | 0.3 | - |
| Haematocrit, % | 1.12 (0.77 – 2.88) | 0.3 | - |
| Total cholesterol, mg/dL | 1.01 (0.99 – 1.01) | 0.2 | - |
| HDL, mg/dL | 0.99 (0.98 – 1.01) | 0.1 | - |
| Triglycerides, mg/dL | 1.01 (0.99 – 1.01) | 0.4 | - |
| Diuretics | 1.75 (0.75 – 4.08) | 0.1 | - |
| SUA*age | 0.99 (0.98 – 1.01) | 0.05 | - |
| SUA*gender (male) | 1.03 (0.95 – 1.15) | 0.1 |  |
| SUA*diabetes mellitus | 0.93 (0.82 – 1.14) | 0.4 | - |
| SUA*eGFR | 0.93 (0.88 – 1.15) | 0.2 | - |
| SUA*diuretics | 1.07 (0.89 – 1.25) | 0.5 | - |

Legend as in the previous tables.

**Supplemental Table 5.** Reclassification analysis investigating the added value of elevated serum uric acid levels (♂ > 5.6 mg/dL; ♀ > 5.1 mg/dL) to different combinations of metabolic syndrome in predicting cardiovascular mortality.

| **MS combination** | **cNRI** | **p-value** | **IDI** | **p-value** |
| --- | --- | --- | --- | --- |
| systemic hypertension + elevated triglycerides + diminished HDL | 8.3% | 0.003 | 5.1% | 0.001 |
| systemic hypertension + elevated triglycerides + elevated fasting glucose | 6.9% | 0.01 | 4.4% | 0.01 |
| systemic hypertension + diminished HDL + elevated fasting glucose | 5.8% | 0.03 | 4.1% | 0.02 |

cNRI: continuous net reclassification improvement; IDI: integrated discrimination improvement; MS: metabolic syndrome.

**Supplemental Table 6.** Correlation analyses between serum uric acid levels and the individual components of metabolic syndrome.

| Variable | Serum uric acid (mg/dL) |
| --- | --- |
|  |  |
| Waist circumference (cm) | 0.26; p<0.0001 |
| Triglycerides (mg/dL) | 0.31; p<0.0001 |
| HDL cholesterol (mg/dL) | -0.24; p<0.0001 |
| Fasting glucose (mg/dL) | 0.41; p<0.0001 |
| Systolic blood pressure (mmHg) | 0.18; p<0.0001 |
| Diastolic blood pressure (mmHg) | 0.17; p<0.0001 |

Values are correlation coefficients.

Legend as in the previous tables.
